# Heart Failure in Patients with Chronic Kidney Disease

**DOI:** 10.3390/jcm12186105

**Published:** 2023-09-21

**Authors:** Andrew Xanthopoulos, Adamantia Papamichail, Alexandros Briasoulis, Konstantinos Loritis, Angeliki Bourazana, Dimitrios E. Magouliotis, Pantelis Sarafidis, Ioannis Stefanidis, John Skoularigis, Filippos Triposkiadis

**Affiliations:** 1 Department of Cardiology, University Hospital of Larissa, 41110 Larissa, Greece; 2 Amyloidosis Center, Department of Clinical Therapeutics, Faculty of Medicine, Alexandra Hospital, National and Kapodistrian University of Athens, 15772 Athens, Greece; 3 Unit of Quality Improvement, Department of Cardiothoracic Surgery, University of Thessaly, 41110 Larissa, Greece; 4 Department of Nephrology, Hippokration Hospital, Aristotle University of Thessaloniki, 54124 Thessaloniki, Greece; 5 Department of Nephrology, Faculty of Medicine, University of Thessaly, 41110 Larissa, Greece; 6 School of Medicine, European University Cyprus, 2404 Nicosia, Cyprus

**Keywords:** chronic kidney disease, heart failure, β-blockers, renin-angiotensin aldosterone system inhibitors, angiotensin receptor/neprilysin inhibitor, sodium glucose cotransporter 2 inhibitor

## Abstract

The function of the kidney is tightly linked to the function of the heart. Dysfunction/disease of the kidney may initiate, accentuate, or precipitate of the cardiac dysfunction/disease and vice versa, contributing to a negative spiral. Further, the reciprocal association between the heart and the kidney may occur on top of other entities, usually diabetes, hypertension, and atherosclerosis, simultaneously affecting the two organs. Chronic kidney disease (CKD) can influence cardiac function through altered hemodynamics and salt and water retention, leading to venous congestion and therefore, not surprisingly, to heart failure (HF). Management of HF in CKD is challenging due to several factors, including complex interplays between these two conditions, the effect of kidney dysfunction on the metabolism of HF medications, the effect of HF medications on kidney function, and the high risk for anemia and hyperkalemia. As a result, in most HF trials, patients with severe renal impairment (i.e., eGFR 30 mL/min/1.73 m^2^ or less) are excluded. The present review discusses the epidemiology, pathophysiology, and current medical management in patients with HF developing in the context of CKD.

## 1. Introduction

Patients with chronic kidney disease (CKD) frequently suffer from heart failure (HF) [1,2]. Management of HF in CKD patients is challenging since several of the routinely used HF medications may further impair renal function or necessitate dosage modifications [3]. Importantly, despite the high prevalence of HF in CKD, and especially in advanced CKD (stages 4–5, kidney replacement therapy) [4], advanced CKD patients are excluded or belittled in most major randomized trials that have shaped the management of HF [5,6]. In this manuscript, following a brief review of epidemiological data and pathogenesis, the advantages, disadvantages, and restrictions of the several pharmaceutical approaches as they apply to the management of HF in patients with underlying CKD are discussed. Unavoidably, several statements in this review are based on pathophysiology and opinion due to the lack of relevant evidence, as previously mentioned.

## 2. Definitions

### 2.1. Chronic Kidney Disease

**Figure 1:**
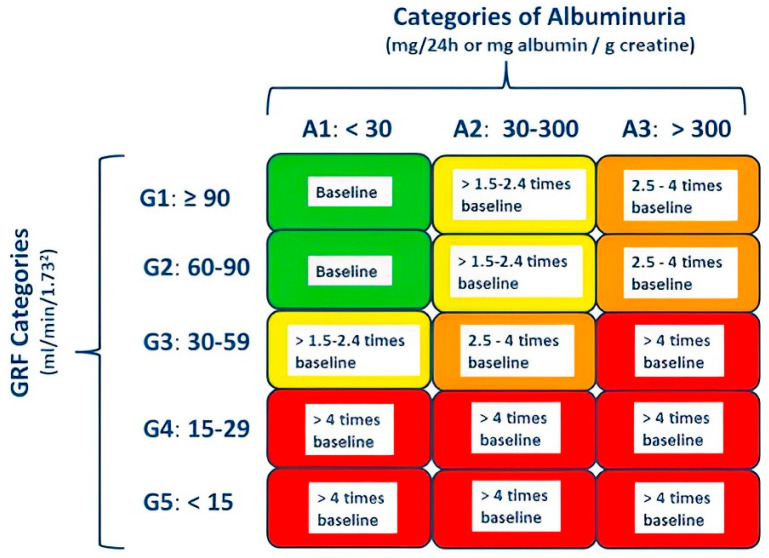
Stages of chronic kidney disease (CKD) according to KDIGO 2012 Clinical Practice Guideline for the Evaluation and Management of Chronic Kidney Disease and cardiovascular mortality risk [7]. CKD can be diagnosed if glomerular filtration rate (GFR) is less than 60 mL/min per 1.73 m^2^ or albuminuria is greater than 30 mg/24 h, both persisting for more than 3 months. Categories are defined by GFR and albuminuria measures (e.g., CKD category G3A3). Green, low risk; yellow, increased risk; orange, high risk; red, very high risk.

According to the National Kidney Foundation Kidney Disease Outcomes Quality Initiative (K/DOQI) guideline, serum creatinine alone must not be used to diagnose CKD since it is not a sensitive marker of glomerular filtration rate (GFR). Assessment for CKD includes measuring estimated GFR (eGFR), urinalysis, and albuminuria quantification [7] (Figure 1). An eGFR less than 60 mL/min/1.73 m^2^ body surface area (BSA) is indicative of CKD, even when evidence of kidney damage such as albuminuria is absent. In patients with eGFR 60 mL/min/1.73 m^2^ or more, kidney damage reflected by the occurrence of albuminuria (30 mg or more albumin/gr creatinine) must be documented before the diagnosis of CKD can be made. A decrease in eGFR or damage of the kidney must be present for more than 3 months for the diagnosis of CKD [8]. 

It is noteworthy that many studies including patients with renal dysfunction are based on eGFR measurements with the Cockcroft–Gault formula, an antiquated equation evaluated in a small patient number [9]. The American National Kidney Foundation endorses the use of the Chronic Kidney Disease Epidemiology Collaboration (CKD-EPI) or the Modification of Diet in Renal Disease (MDRD) equations, which are more precise than the Cockcroft–Gault equation [8]. Drug dosage must usually be decreased in CKD proportionally to the estimated reduction in the active drug clearance. In adjusting drug dosage, patient-related factors include the severity of renal dysfunction and patients’ size, whereas factors related to the drug include the drug fraction that is excreted unchanged in the urine and drug therapeutic window [10]. 

### 2.2. Acute Kidney Injury

Acute kidney injury (AKI) is classified in three stages [11]: (a) Stage 1: creatinine increases 1.5 times or more compared with baseline or 0.3 mg/dL or more within a 48 h period, or the urine volume decreases to less than 0.5 mL/kg for 6–12 h; (b) Stage 2: creatinine increases 2.0 times or more compared with baseline, or the urine volume decreases to less than 0.5 mL/kg for 12 h or more; and (c) Stage 3: creatinine increases 3.0 times or more compared with baseline or increases to 4.0 mg/dL or more, or renal replacement therapy is indicated, or the urine volume decreases to less than 0.3 mL/kg for 24 h or more. Most AKI cases settle within 7 days [12]. However, if they are sustained or relapse, the clinical outcome is precarious. Patients in AKI stage 2–3 recuperating within one week and leaving the hospital without renal impairment have a one-year survival of 90% or more, whereas those who are not recovering have a 47% mortality during hospital stay, and among those leaving the hospital, the survival at one year is approximately 77% [12].

## 3. Epidemiology

CKD patients often suffer from cardiovascular (CV) disease (CVD). Approximately 50% of CKD patients in stages 4 and 5 suffer from CVD [13], and CV mortality underlies approximately 40–50% of total mortality in CKD stages 4 (advanced kidney disease) and 5 (end-stage kidney disease) [14,15]. Besides the high risk for fatal myocardial infarction and stroke, CV death may also result from malignant arrhythmias and HF, especially in advanced CKD. New-onset HF in CKD patients occurs in the range of 17–21% [16]. A decrease in the eGFR and/or an increase in the urine albumin-to-creatinine ratio are accompanied by a higher risk for new onset HF [17]. HF prevalence increases with declining renal function, reaching approximately 45% in patients undergoing dialysis, with half of them having a reduced left ventricular (LV) ejection fraction (LVEF) [18]. The prognosis of CKD patients with HF is poor and worsens with deteriorating renal function [19]. In most patients, the coexistence of CKD and HF is due to common risk factors such as diabetes, hypertension, and atherosclerosis.

## 4. Mechanisms of HF Development in CKD

**Figure 2:**
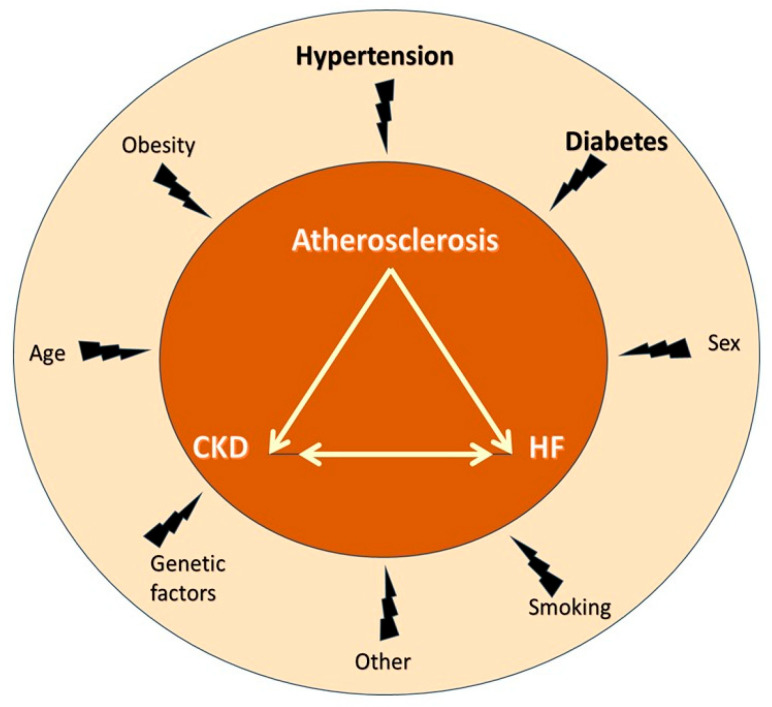
The “cardiovascular death triangle” comprised of atherosclerosis, chronic kidney disease, and heart failure and associated risk factors. Notably, atherosclerosis, chronic kidney disease, and heart failure share many common risk factors, the most important being hypertension and diabetes. Abbreviations: CKD, chronic kidney disease; HF, heart failure.

The kidney and the heart are important for CV homeostasis. In healthy individuals, renal hemodynamics affect cardiac hemodynamics and vice versa, and this reciprocal interaction is finetuned by neurohormonal activity, including the natriuretic peptide system, the renin–angiotensin aldosterone system (RAAS), and the sympathetic nervous system (SNS) [20]. Dysfunction/disease of the kidney may precipitate dysfunction/disease of the heart and vice versa, contributing to the development of a negative spiral. Further, the reciprocal kidney–heart interaction often occurs on top of diseases involving both organs at the same time, usually diabetes and hypertension, both major atherosclerosis risk factors (Figure 2).

HF in CKD develops as result of a CKD-induced systemic low-grade inflammation, bringing about vascular and myocardial remodeling, which in turn leads to hypertension, atherosclerosis, vascular calcification, and vascular senescence as well as valvular calcification and myocardial fibrosis [21]. The progression of CKD following HF development is driven by hemodynamic, neurohormonal, and CVD-related processes [22]. Hemodynamic processes (decreased cardiac output and increased systemic venous pressure) further increase salt and fluid retention, augmenting systemic and renal congestion as well as renal interstitial compression [23], thus accelerating renal dysfunction. Neurohormonal mechanisms comprise RAAS and SNS overactivity, whereas CVD-related processes involve multiple pathways that instigate progression of CKD, including the aggravation of systemic and local inflammatory processes and altered immune responses [24].

**Figure 3:**
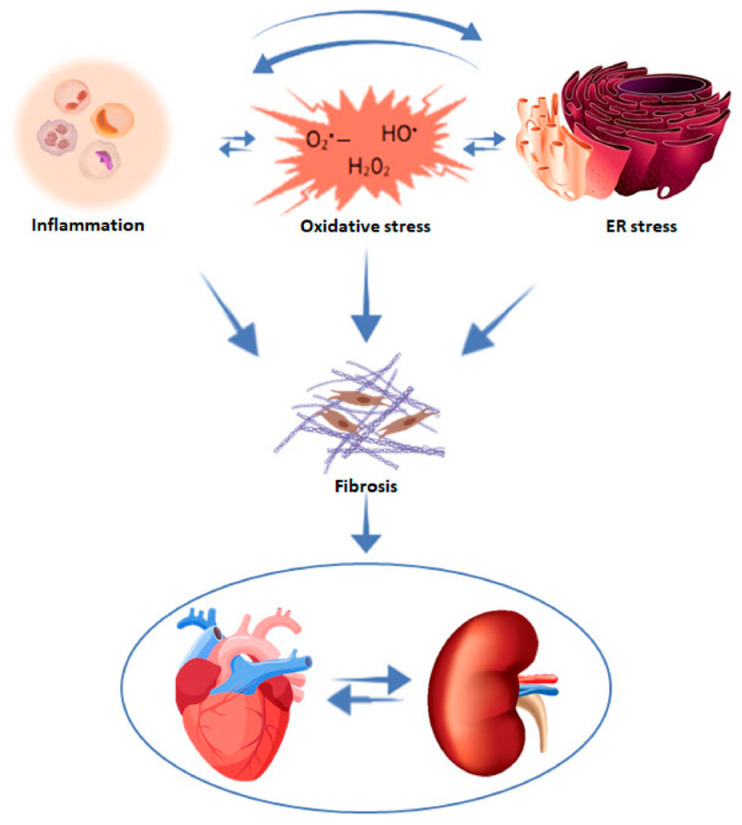
Mechanisms contributing to cardiac and renal fibrosis. With permission from ref. [26]. Abbreviation: ER stress, endoplasmic reticulum stress.

Fibrosis is a unifying pathophysiology of renal and cardiac dysfunction (Figure 3) [25,26]. Injury of an organ initiates a complex cascade of cellular and molecular processes culminating in tissue fibrosis. Despite the fact that this fibrogenic response may be initially adaptive, its prolongation may cause parenchymal scarring and ultimately cellular dysfunction and organ failure [27]. Both in the kidney and the heart, fibrosis is the common denominator of neurohormonal overactivity, inflammation, and endothelial dysfunction due to oxidative stress [28], eventually leading to CKD, CVD, and HF.

Finally, both CKD and HF are frequently accompanied by anemia, which is due to a constellation of diverse factors, including relative erythropoietin deficiency, uremia-induced inhibitors of erythropoiesis, short erythrocyte survival, and disturbed iron homeostasis [29]. The decrease in endogenous erythropoietin together with the anemia-related reduced oxygen transport aggravates tissue hypoxia and neurohormonal overactivity, facilitating the further deterioration of renal and cardiac function [30].

## 5. Major Limitation of Cardiovascular Trials: The Exclusion of Patients with Renal Disease

In most CV trials, CKD patients are excluded based on diverse criteria (e.g., serum creatinine 1.5 mg/dL or more, 2.3 mg/dL or more, 3 mg/dL or more, or eGFR less than 30 mL/min/1.73 m^2^), belittling CKD patients [4,25]. CKD patients in stages 4 and 5 have been excluded from HF trials due to worries that potential drug accumulation may cause complications. Exclusion of CKD patients is particularly widespread in trials evaluating β-blockers, RAAS inhibitors (RAASi), and angiotensin receptor/neprilysin inhibitors (ARNI), limiting evidence to support or not the use of these agents in CKD patients with HF [31]. Clinical research must be promoted in patients with combined renal and cardiac diseases to achieve evidence-based management and avoid complications [25].

## 6. Medical Treatment of HF in CKD

Five well-known drug categories are considered the pylons of the treatment of chronic HF [1]. These include (1) β-blockers; (2) RAASi: angiotensin converting enzyme inhibitors (ACEi), angiotensin receptor blockers (ARB), and mineralocorticoid receptor antagonists (MRA); (3) ARNI: sacubitril/valsartan; (4) SGLT-2i; and (5) diuretics.

### 6.1. β-Blockers

β-Blockers reduce blood pressure in CKD attenuating the hyperactive SNS [32]. Cardioprotection with these agents has been convincingly documented [33], but they are also extremely useful in CKD patients. Experimental studies have demonstrated renoprotection with β-blockers, including a decrease in interstitial fibrosis after renal injury [34,35]. Further, several studies have demonstrated prolongation of survival with β-blocker therapy in CKD patients [36,37]. Despite these encouraging reports, β-blockers have been underused in CKD patients [38], most likely due to worries regarding control of blood sugar, diminished excretion from the kidneys, and systemic accumulation [39,40]. However, β-blockers can be safely used in all stages of CKD, provided relevant dose adjustments and use of those excreted by the liver that possess ancillary vasodilatory properties, such as carvedilol [41]. Oral clearance of the two enantiomers of nebivolol, another vasodilating β-blocker, is reduced in CKD but is restored with hemodialysis [42]. Compared with ACEi, β-blockers seem to be inferior regarding renoprotection [43,44]. In the African American Study of Kidney Disease and Hypertension (AASK), ramipril proved superior to metoprolol in the retardation of renal dysfunction and mortality reduction in CKD patients [45].

β-blockers have proved beneficial in CKD patients with HF. In a retrospective cohort of elderly CKD patients with HF, β-blockers reduced all-cause mortality, even those with an eGFR 30 mL/min per 1.73 m^2^ or less [46]. Likewise, in another observational study that recruited HF patients with advanced CKD (eGFR 30 mL/min per 1.73 m^2^ or less) from the Swedish Heart Failure Registry, β-blockers use was accompanied by lower morbidity and mortality [47]. As neither of these studies was specifically designed to evaluate the interaction between treatment benefit and LVEF and until a relevant large, randomized control study is conducted, we believe that in the absence of contraindications, patients with CKD and HF should be treated with β-blockers, preferably carvedilol. In this regard, a randomized study in 114 dialysis patients with dilated cardiomyopathy (carvedilol group n = 88 and placebo group n = 56) revealed significantly lower mortality and hospitalizations among patients receiving carvedilol than among those receiving a placebo at 2 years [36]. Furthermore, the active treatment group (carvedilol) exhibited smaller cavity diameters and higher LVEF compared to placebo.

### 6.2. Renin–Angiotensin–Aldosterone System Inhibitors (RAASi)

#### 6.2.1. Angiotensin Converting Enzyme Inhibitors/Angiotensin Receptor Blockers

In mild or moderate CKD, ACEi/ARB reduce blood pressure, retard eGFR decline, attenuate proteinuria, and delay progression to CKD stages 4 or 5 [48,49]. However, ACEi/ARB are often discontinued, especially in patients with a lower eGFR. Hyperkalemia (potassium level 5.3 mEq/L or more), hypotension (systolic blood pressure 90 mmHg or less), low bicarbonate level (less than 22 mmol/L), and hospitalization (usually due to AKI) increase the risk of ACEi/ARB discontinuation [50]. Further, in advanced CKD, a recent study demonstrated that during a follow-up of three years, the frequency of CV events and death was similar in patients who continued vs. those who discontinued ACEi/ARB, indicating that ACEi/ARB may not be as helpful in patients with advanced and progressive CKD [51].

ACEi and ARB differ in their structure and mechanism(s) of action, exhibiting diverse pharmacokinetic properties. The pharmacokinetics of ACEi are poorly characterized owing to several factors interfering with their analysis, whereas the pharmacokinetics of ARB vary the least with renal dysfunction [52,53]. Surprisingly, a network meta-analysis demonstrated that ACEi is superior to ARB and other antihypertensive agents in improving outcomes in non-dialysis CKD 3–5 patients [54].

Regarding HF management, both ACEi and ARB reduce morbidity and mortality [4,55,56]. A study that compared the effectiveness of ACEi and ARB in patients with prior myocardial infarction (MI) reported that during a 3-year follow-up, incident clinical CV outcomes among older patients with MI were lower with ARB compared with ACEi [57]. 

In summary, the choice between ACEi and ARB in patients with CKD and HF should be individualized based on the clinical setting, availability, and healthcare provider expertise [58]. ACEi/ARB may also be used in stage 4–5 CKD, starting at a low dose and carefully monitoring renal function and potassium levels [18].

#### 6.2.2. Mineralocorticoid Receptor Antagonists (MRAs)

Aldosterone attaches to the mineralocorticoid receptor (MR) at the distal nephron epithelium and augments sodium reabsorption and potassium secretion [59]. Inhibition of the MR by an MRA decreases aldosterone-induced sodium and water reabsorption [60], whereas MR inhibition in CV cells (e.g., cardiomyocytes and fibroblasts) as well as in those belonging to the innate and adaptive immunity systems initiates potent beneficial processes with anti-hypertrophic, antifibrotic, and anti-inflammatory effects, all improving cardiac function [61].

Patients treated with ACEi or ARB may exhibit the so-called aldosterone breakthrough, a rise in plasma aldosterone, the prevalence of which in CKD patients can reach 50% [62]. The increased aldosterone levels promote target organ damage on top of volume expansion and hypertension. Aldosterone breakthrough is accompanied by lower plasma renin activity (PRA), which is expressed as a rise in the aldosterone-to-renin ratio (more than 3 ng/dL per ng/mL/h) and reflects the volume expansion caused by aldosterone [63,64]. Several studies have shown that in CKD, the non-selective MRAs (spironolactone or eplerenone), when given on top of ACEi or ARBs, reduce proteinuria but increase potassium levels, and these agents may be beneficial in dialysis patients [65,66,67]. However, a small trial in dialysis patients showed no benefit [68].

Several studies, including the Randomized Aldactone Evaluation Study (RALES) [69], the Comparison Of Outcomes In Patients In New York Heart Association (NYHA) Class II Heart Failure When Treated With Eplerenone Or Placebo In Addition To Standard Heart Failure Medicines (EMPHASIS-HF) [70], and the Treatment of Preserved Cardiac Function Heart Failure With an Aldosterone Antagonist (TOPCAT) [71], have demonstrated that non-selective MRAs are effective in the treatment of HF despite an increase in the potassium levels and in the risk of incident hyperkalemia. A secondary analysis of RALES that investigated the efficacy of spironolactone in patients with severe HF and reduced kidney function reported that spironolactone was associated with a reduction in all-cause mortality and hospital stays for HF regardless of baseline kidney function [72]. In the same study, worsening kidney function increased mortality in the placebo but not in the aldosterone group, whereas the risk of hyperkalemia was higher in patients of the aldosterone group and especially those with worse kidney function or worsening kidney function. Thus, non-selective MRAs may be a useful treatment option for patients with reduced kidney function and advanced HF, but careful monitoring of electrolytes is necessary to prevent adverse events.

Finerenone, a novel selective MRA, possesses chemical and physical properties providing balanced renal and cardiac drug delivery [73]. Finerenone exhibited significant renoprotective and cardioprotective effects in two recent clinical trials that recruited patients with CKD and type 2 diabetes mellitus (T2DM). The Finerenone in Reducing Kidney Failure and Disease Progression in Diabetic Kidney Disease (FIDELIO-DKD) study, which recruited patients with T2DM and either CKD in stage 2 to 4 and moderate albuminuria or CKD in stage 1 and 2 with severe albuminuria, reported that finerenone retarded CKD progression and improved outcomes [74]. Similar were the findings in the Finerenone in Reducing Cardiovascular Mortality and Morbidity in Diabetic Kidney Disease (FIGARO-DKD) trial [75]. It is noteworthy that the patients of both studies had a high risk of hyperkalemia (coexistence of T2DM, RAASi treatment, and advanced CKD in some patients), as indicated by the high incidence of hyperkalemia in the relevant placebo groups. Nevertheless, despite the higher incidence of hyperkalemia in the finerenone than in the placebo group, treatment interruption due to hyperkalemia was infrequent, and no death related to hyperkalemia was reported [76]. In conclusion, finerenone provides robust renoprotection and safety across the spectrum of CKD in T2DM [77] and can be used as an early treatment to slow CKD progression in T2DM patients and most likely in those without. Regarding HF, although finerenone has demonstrated consistent anti-inflammatory/anti-fibrotic effect and beneficial actions on cardiac hypertrophy, diastolic dysfunction, and LV systolic dysfunction in experimental models, relevant clinical studies evaluating the effect of finerenone in HF are lacking [78]. However, in a meta-analysis of three trials with 1520 HF patients, finerenone improved several biochemical indicators, including biomarkers of cardiac and renal function [79].

In summary, the non-steroidal MRAs (spironolactone and eplerenone) are currently the agents of choice in CKD patients with HF, although finerenone is a very promising agent with attractive properties. However, there is lack of evidence regarding the safety and efficacy of MRA in patients with eGFR 30 mL/min/1.73 m^2^ or less.

### 6.3. Sacubitril-Valsartan

Sacubitril/valsartan attenuates the neurohormonal overactivity in HF by inhibiting both the neutral endopeptidase neprilysin (sacubitril) and the angiotensin II type 1 receptors (valsartan), therefore improving the neurohormonal balance more than RASi [80]. 

Sacubitril/valsartan has proved beneficial in CKD patients. In the United Kingdom Heart and Renal Protection-III (UK HARP-III) trial (n = 414; eGFR: 20 to 60 mL/min/1.73 m^2^; follow-up: 12 months), although sacubitril/valsartan and irbesartan had similar effects on renal function and albuminuria, sacubitril/valsartan proved superior to irbesartan in lowering blood pressure and cardiac biomarkers [81]. Further, sacubitril/valsartan has an important role in the treatment of patients with HF, as demonstrated in the Prospective Comparison of ARNI with ACEI to Determine Impact on Global Mortality and Morbidity in Heart Failure (PARADIGM-HF) [82] and the Prospective Comparison of ARNI With ARB Global Outcomes in HF With Preserved Ejection Fraction (PARAGON-HF) [83] trials.

Sacubitril/valsartan is beneficial in patients with coexistent CKD and HF. A meta-analysis of three randomized control trials (RCTs) with 3460 patients with CKD and HF compared sacubitril/valsartan with irbesartan, valsartan, and enalapril [84]. Sacubitril/valsartan significantly increased eGFR but did not reduce the urinary albumin/creatinine ratio more than control. Further, sacubitril/valsartan reduced systolic blood pressure and NT-proBNP more than the control, whereas no significant difference between sacubitril/valsartan and control regarding the side effects was observed. Similar were the findings in another meta-analysis of 10 RCTs that included 16,456 patients with CKD and HF [85]. Sacubitril/valsartan resulted in a lower risk of renal dysfunction as compared with ACEi/ARB alone. Finally, in a recent study, sacubitril/valsartan was tested in coexistent end-stage kidney disease and HF with reduced LVEF. After one year, LV systolic and diastolic function improved with sacubitril/valsartan but were unchanged with conventional treatment [86].

In summary, sacubitril/valsartan is extremely useful in lessening cardiac events and ameliorating renal impairment in CKD patients with HF.

### 6.4. Sodium-Glucose Cotransporter 2 Inhibitors (SGLT-2i)

The SGLT-2i were initially considered glucose-lowering agents. However, due to their pleiotropic effects, the use of SGLT-2i has expanded far beyond T2DM and nowadays includes CKD and HF with or without diabetes. 

The mechanisms underpinning the renal and cardiovascular benefits of SGLT-2i are multiple, with some related and some unrelated to the hypoglycemic effect of these agents [87]. SGLT-2i prevent both hyperglycemia and hypoglycemia, with a slight effect on HbA1C, decreasing fat mass and refining glomerular hemodynamics, thereby attenuating albuminuria and O_2_ and the need for tubular reabsorption and increasing cortical oxygenation, which, in association with the diminished tubular glucotoxicity, protect tubular function and maintain GFR. Further, it seems that SGLT-2i imitate systemic hypoxia and prompt erythropoiesis, lessen volume retention and blood pressure, and preserve CV function, presumably by conquering diuretic and natriuretic-peptide resistance and hampering Na+-H+ exchangers and sympathetic tone [88].

The first trial assessing the SGLT-2i effect on renal dysfunction was the Canagliflozin and Renal Events in Diabetes with Established Nephropathy Clinical Evaluation (CREDENCE) study (4401 diabetic patients with eGFR 30 to less than 90 mL/min per 1·73 m^2^ and albuminuria more than 300 to 5000 mg/g), which was stopped early, as canagliflozin reduced by 30% the primary outcome (end-stage kidney disease, doubling of the serum creatinine level, or death from renal or CV causes) [89].

The renoprotective effects of SGLT-2i have also been documented in patients without diabetes. The Dapagliflozin and Prevention of Adverse Outcomes in Chronic Kidney Disease (DAPA-CKD) trial (4304 participants with an eGFR 25–75 mL per minute per 1.73 m^2^ and urinary albumin-to-creatinine ratio 200 to 5000 mg/g) reported that dapagliflozin reduced the risk of the primary endpoint (decline eGFR of 50% more, end-stage kidney disease, or death from renal or CV causes) by 44% compared with placebo regardless of diabetes status or CKD cause [90]. Similar were the findings in the recent Empagliflozin in Patients with Chronic Kidney Disease (EMPA-KIDNEY) trial (6609 patients with CKD, eGFR of 20 to 45 mL/min/1.73 m^2^, or eGFR of 45 but less than 90 mL/min/1.73 m^2^ plus urinary albumin-to-creatinine ratio of 200 mg/g or more), in which empagliflozin reduced the primary endpoint (end-stage kidney disease, eGFR 10 mL/minute/1.73 m^2^ or less, a sustained decrease in eGFR of 40% or more from baseline, or death from renal causes) compared with placebo [91]. 

There is compelling evidence that SGLT-2i also improve outcomes in HF as reported by several trials, including the Dapagliflozin and Prevention of Adverse Outcomes in Heart Failure (DAPA-HF) [92], Empagliflozin Outcome Trial in Patients with Chronic Heart Failure and a Reduced Ejection Fraction (EMPEROR-Reduced) [93], Empagliflozin Outcome Trial in Patients with Chronic Heart Failure with Preserved Ejection Fraction (EMPEROR-Preserved) [94], the Dapagliflozin Evaluation to Improve the Lives of Patients with Preserved Ejection Fraction Heart Failure (DELIVER) [95], and Empagliflozin in Patients Hospitalized for Acute Heart Failure (EMPULSE) [96]. 

Based on the above, the benefits of SGLT-2i are consistent across many CKD with coexistent HF subgroups regardless of the T2DM status [97]. In addition, SGLT-2i reduce new-onset anemia and hyperkalemia in patients with CKD and are safe and generally well tolerated. Although there are few data on SGLT-2i for end-stage kidney disease and transplant recipients, many studies have started to enroll patients with severely impaired kidney function, defined as eGFR 25 mL/min/1.73 m^2^ or less [98]. One of the limited number of studies including diabetic kidney transplant recipients reported that SGLT-2i use was associated with improvement in weight as well as hypomagnesemia, without significant changes in renal function at 6 months [99].

### 6.5. Diuretics

Diuretics are of prime importance in the management of volume overload and comprise several classes [100]. Loop diuretics are the agents of choice for combating volume overload. They inhibit the Na+/K+/2Cl- symporter (NKCC2) in the thick ascending loop of Henle, thereby preventing reabsorption of tubular Na+ and eventually leading to Na+ and water removal [101]. Loop diuretics are initially tackled by organic anion transporters (OATs) and afterwards secreted into the luminal surface of the renal tubule where their action takes place. In addition, they may cause venous dilation, leading to a reduction in venous return and cardiac preload with consequent relief of dyspnea even before initiation of diuresis.

Water and sodium retention, often resulting from an unjustified interruption or reduction of doses by the patients themselves, lead to venous congestion associated with dyspnea on the one hand and worsening renal function on the other. Diuretics cause decongestion but may worsen renal function since the optimal stopping point for decongestive therapy and avoidance of hypovolemia remain major challenges in HF management. Larger doses of diuretics are required in patients with CKD and HF, as diuretic resistance is frequent in this context [102]. Many mechanisms contribute to diuretic resistance, including reduced gastrointestinal tract absorption, diminished plasma albumin, neurohormonal activation, nephrotic syndrome, and hypotension [102].

Practical approaches to overcome diuretic resistance include readjustment of the diuretic dose; addition of other categories of diuretics (preferably thiazides), always with caution regarding electrolyte disturbances; change from furosemide to torasemide due to its long action; and discontinuation of anti-inflammatory drugs [102]. The recent Acetazolamide in Decompensated Heart Failure With Volume Overload (ADVOR) study showed that the addition of 500 mg of acetazolamide (a carbonic anhydrase inhibitor) daily to loop diuretics increased the rate of successful decongestion [103]. In a large observational study (≈11,000 patients) of hemodialysis patients, the use of loop diuretics reduced hospitalizations and hypotensive episodes during hemodialysis but not mortality at one year [104].

Loop diuretics are indicated to alleviate congestion in symptomatic patients, and when this is achieved, the dose has to be down-titrated to the lowest dose that will keep the patient euvolemic. Higher stages of CKD may require higher doses of loop diuretics to reach decongestion or euvolemia, as tubular delivery of diuretics decreases [4,103]. It is noteworthy, that loop diuretics can utilize residual renal function and attenuate excess fluid retention that can cause congestion and hyperkalemia, the two most frequent indications for emergency dialysis [105]. Finally, although diuretic use does not appear to attenuate the benefit from SGLT-2i in preventing adverse kidney events or AKI, renal function and electrolytes should be closely monitored, especially during loop diuretic initiation or after modifications in loop diuretic dose [106]. 

## 7. Management of Specific Conditions

### 7.1. Worsening Renal Function

Worsening renal function (WRF) is usually defined as a 20–30% decrease in eGFR or an increase in creatinine of 0.3 mg/dL or more 15–30 days after an intervention and occurs in approximately 25% of the cases [107,108]. The prognostic significance of WRF depends on the context in which it develops. WRF appearing after initiation or up-titration of treatment with β-blockers may reflect a decrease in cardiac output and necessitates a thorough re-evaluation of hemodynamics and clinical status, as it is accompanied by a worse prognosis [109]. In contrast, RAASi, sacubitril/valsartan, and SGLT-2i lessen pressures within the glomerulus accompanied by acute eGFR reduction, which reflects a rearrangement of renal hemodynamics, and it is not linked to a decrease in the number of nephrons that function. Usually, the eGFR slope reaches a plateau after the acute phase, and the subsequent curve inversion is indicative of a slower CKD progression [110]. Renal function should be tested during initiation and titration of RAASi. A decline in renal function of 30% is allowable. According to the Heart Failure Working Group of the French Society of Cardiology, the following should be considered [110]:

(A) After RAASi initiation, rise in creatinine up to 50% and a rise in potassium to 5.5 mmol/L or less are allowable;

(B) A rise in creatinine 50–100% requires a systematic evaluation of the clinical context (congestion, dehydration, blood pressure, and concomitant interaction) and adjustment of medications (halving of usual dose may be considered). Close monitoring of the patient is also required;

(C) A rise of potassium more than 5.5 mmol/L or a rise in creatinine more than 100% or a creatinine level more than 310 mol/L (3.5 mg/dL) or eGFR less than 20 mL/min/1.73 m^2^ necessitates discontinuation of RAASi and ARNI and seeking the advice of a nephrologist;

(D) A rise in creatinine more than 100% or underlying CKD with an acute decrease in eGFR to less than 20 mL/min/1.73 m^2^, acutely decompensated HF, severe ionic disorders (e.g., hyponatremia (sodium level less than 125 mEq/L), hypokalemia (potassium level less than 3 mEq/L) or hyperkalemia (potassium level more than 6.0 mEq/L)), severe dehydration with symptomatic hypotension, cardiogenic shock, hemodynamic instability accompanied by urinary tract disorders (obstruction/infection), or ineffectiveness of 48 h outpatient treatment require urgent hospital referral.

### 7.2. Hyperkalemia

Hyperkalemia, defined as a serum potassium level > 5.0 to 5.5 mmol/L, is uncommon in health due to the renal excretion of the dietary potassium, but it is one of the most prevalent electrolyte disorders in CKD, adversely affecting outcomes [111]. The prevalence of hyperkalemia rises in the advanced CKD stages and is linked to poor prognosis. The relationship between potassium intake and serum potassium levels is virtually unknown, and this has generated controversy regarding the correct nutritional approach to hyperkalemia in CKD patients [112]. Another issue is that RAASi-induced hyperkalemia is frequently associated with an unjustified down-titration or discontinuation of these agents in CKD patients, depriving them of major renoprotective and cardioprotective interventions that prolong survival [113]. Fortunately, nowadays, patiromer and sodium zirconium cyclosilicate (SZC) allow clinicians to maintain patients on RAASi and up-titrate medications to optimal doses without worrying for hyperkalemia and its adverse effects [113,114].

Timely detection of moderate or severe hyperkalemia is of major importance to avoid fatal cardiac arrhythmias and paralysis of the muscles. Treatment of hyperkalemia entails the eradication of reversible etiology (diet and medications) and implementation of therapies that act rapidly by shifting potassium into cells and mitigate the effects of hyperkalemia on cardiac membrane and measures augmenting the removal of potassium from the organism (e.g., saline, resins, and hemodialysis). Hyperkalemia with electrocardiographic (ECG) changes or with a potassium level more than 6.5 mEq/L (even in the absence of ECG changes) should be treated as a medical emergency (Table 1).

Treatment must be started with calcium gluconate for cardiomyocyte membrane stabilization, followed by insulin injection and β-agonist administration. During intercurrent illness, stopping RAASi should be discouraged, but if potassium levels exceed 6.0 mEq/L, or creatinine rise exceeds 30%, RAASi should be temporarily discontinued [115]. In the presence of fluid retention, high doses of diuretics may be needed, and a deterioration of renal function is not an indication to decrease diuretic dosage. In contrast, if the patient remains congested, an increase in diuretic dose may be required. In hypovolemic patients, diuretics should be stopped for a short time. Treatment with RAASi should be initiated, up-titrated, and maintained as soon as possible whether during intercurrent illness or worsening HF [116].

### 7.3. Anemia

**Figure 4:**
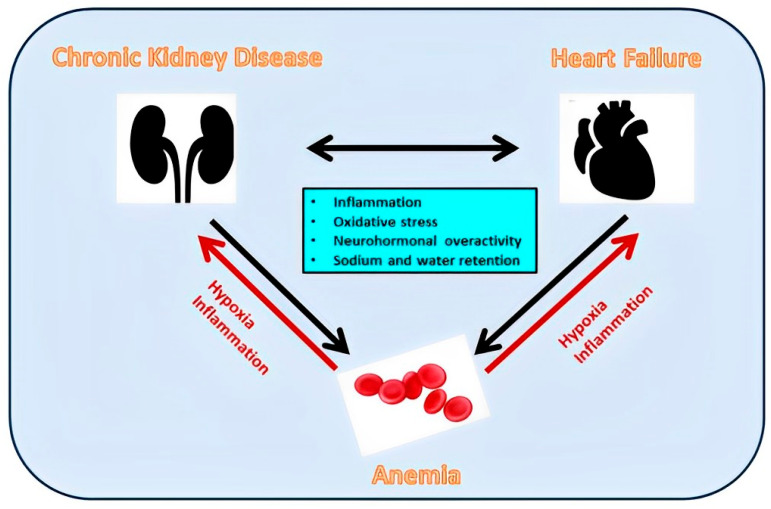
Inflammation, oxidative stress, neurohormonal overactivity, and sodium and water retention contribute both to adverse renocardiac interactions and anemia development. The latter contributes to a further deterioration of renal and cardiac function.

The constellation of CKD, HF, and anemia (Figure 4) occurs frequently and carries high morbidity and mortality [117]. Although anemia is associated with increased risk, intensive anemia treatment with erythropoietin (EPO) and subsequently with other erythropoiesis-stimulating agents (ESA) is of limited usefulness [118,119,120]. Possible explanations are either that the associations of anemia with CKD and HF prognosis do not reflect direct causality but residual confounding or reverse causality, or the use of EPO/ESA increases risk through other side effects outside of erythropoiesis [121]. Based on the above, interest is shifted to iron-replacement therapy since at least every second patient with CKD [118] or with CHF [122] is iron-deficient with or without anemia.

Patients with advanced CKD and iron deficiency should be treated with iron supplementation, as first demonstrated in the Proactive IV irOn Therapy in hemodiALysis (PIVOTAL), which demonstrated that high-dose intravenous iron was superior to the low-dose regimen and resulted in lower doses of ESA being administered [123]. Similar findings have been reported with intravenous iron in HF patients [124].

A turning point in the management of anemia both in CKD and HF has been treatment with SGLT-2i. These agents, which favorably affect prognosis both in CKD and HF, increase hemoglobin by approximately 0.6–0.7 g/dL, an effect that has been linked to a rise in EPO and an expansion in red blood cell mass that alleviate anemia [125]. The extent of this effect can be compared to that of low to medium doses of hypoxia-inducible factor (HIF)/prolyl hydroxylase domain (PHD) inhibitors, which have been developed as a potential alternative to EPO/ESA agents [30]. Interestingly, HIF/PHD inhibitors act by preventing degradation and increasing the levels of both isoform HIF-1α and isoform HIF-2α [126]. However, only the HIF-2α isoform stimulates the production of EPO, and upregulation of HIF-1α may be an unnecessary ancillary property of HIF-PHD inhibitors. In contrast, SGLT-2i selectively increase HIF-2α and downregulate HIF-1α, a distinctive property that may contribute to their renoprotective and cardioprotective effects. Thus, SGLT-2i should be evaluated as a treatment option for anemia both in CKD and HF.

## 8. Conclusions-Perspective

**Figure 5:**
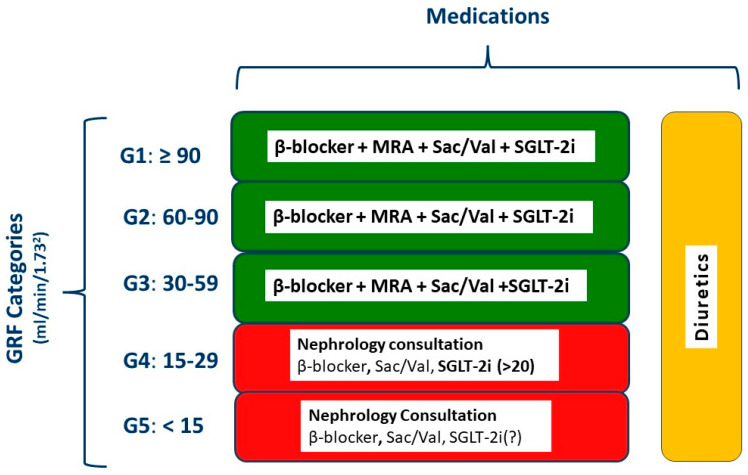
Management of heart failure according to the stage of chronic kidney disease. GFR, glomerular filtration rate; MRA, mineralocorticoid receptor antagonist; Sac/Val, sacubitril/valsartan; SGLT-2i, sodium glucose cotransporter 2 inhibitor; ACEi, angiotensin converting enzyme inhibitor; ARB, angiotensin receptor blocker. Red, advanced renal disease necessitating nephrology consultation.

CKD affects millions of individuals globally, and for the majority of CKD, patients the risk of developing CVD is higher than the risk of progression to advanced or end-stage kidney disease. Further, CV mortality in CKD patients is markedly higher than in the general population, and LV ventricular hypertrophy as well as HF contribute to the grave prognosis. The etiology of CV complications and especially HF in CKD is multifactorial. SGLT-2i have revolutionized treatment of CKD complicated by HF (Figure 5). The beneficial effects of SGLT-2i are diverse and include stimulation of erythropoiesis and attenuation of anemia, which is a common and severe complication in this setting. Considering that anemia treatment in the context of CKD and HF is challenging due to the multiple interactions and pathophysiologic complexity, evaluation of SGLT-2i as antianemia drugs with an appropriately defined treatment target has become mandatory.

## Figures and Tables

**Table 1:** Management of hyperkalemia.

Agent	Mechanism of Action	Dose	Side Effects
Mild/Moderate Hyperkalemia (Serum K+ > 5 mEq to <6 mEq/L without ECG changes)			
Sodium polystyrene sulfate (SPS): Oldest of potassium binding resins. Use limited by side effects.	SPS binds to K^+^ in the intestine in exchange for Na^+^.	SPS oral: 15 g 1–4 times daily. SPS rectal: 30–50 g every 6 h. The 30 g dose lowers K^+^ by ≈1 mEq/L. Action appears at 2–6 h.	Nausea Vomiting Constipation Diarrhea
Patiromer: K^+^ binder	It is nonabsorbable, binds more K^+^ than SPS and exchanges K^+^ for Ca++ and therefore is suitable for patients with heart failure.	The effect appears 4–7 h from first dose. *Initial dose*: 8.4 g orally once daily. Serum K^+^ should be monitored and dose adjusted in 8.4 mg increments at 1-week intervals depending on serum K^+^ level and target range. *Maintenance dose*: 8.4 to 25.2 mg/day. Maximum dose 25.2 g/day. -All medications should be spaced apart by 3 h from patiromer.	Hypomagnesemia Constipation Flatulence Diarrhea
Sodium zirconium cyclosilicate (SZC): K^+^ binder	Insoluble compound working throughout the gastrointestinal tract by binding K^+^ and exchanging it for Na^+^ and H^+^.	*Initial dose*: 10 g orally 3 times a day for up to 48 h, then 10 g orally once daily. *Maintenance dose*: 5 g every other day to 15 g once a day	Hypertension Peripheral edema Urinary tract infections
**Severe Hyperkalemia** **(Serum K+ > 5 mEq/L to < 6.0 mEq with ECG changes** **or** **K+ ≥ 6.0 mEq/L (even without ECG changes))**			
Calcium: Rapid response. Intravenous (IV) Ca++ salts should be administered immediately in hyperkalemic patients presenting with electrocardiographic (ECG) changes suggesting hyperkalemia. Ca++ is also indicated when K^+^ > 6.5 mEq/L regardless of the presence or absence of ECG changes.	Cardiomyocyte protection. Membrane stabilization with Ca++ is essential due to the cardiotoxic effects of hyperkalemia. Ca++ does not reduce the K^+^ level and must be combined with potassium-lowering interventions.	*Calcium chloride*: 0.5–1 g IV over 2–5 min. The effect appears within 1–2 min and lasts 30–60 min. *Calcium gluconate*: 1–3 g IV over 2–5 min. The effects appear within 5 min, and the dose can be repeated at this interval in cases with sustained, life-threatening ECG changes.	Hypotension Bradycardia
Insulin: Intermediate response	Intracellular shift of K^+^. Insulin acts on the glucose transporter type 4 promoting intracellular movement of potassium through the Na^+^/K^+^ ATPase pump.	Ten-unit bolus of regular insulin IV together with 1 ampule of 25 g dextrose to prevent the hypoglycemic effects. IV insulin lowers the serum potassium level by ≈1 mEq/L. The effect appears within 10–20 min and lasts about 4–6 h.	Hypoglycemia
Salbutamol: Intermediate response	Intracellular shift of K^+^. Salbutamol is a β2 agonist that also activates the Na^+^/K^+^ ATPase transporter on muscle and liver cells.	Amount of 10–20 mg of nebulized salbutamol will lower the K^+^ by 0.5 to 1.0 mEq/L. The effect appears within 15–30 min and lasts at least 2 h.	Trembling Palpitations
Sodium bicarbonate: Intermediate response. Only in patients with metabolic acidosis or in the setting of cardiac arrest.	Intracellular shift of K_+_ by serum alkalinization, and direct bicarbonate transport into muscle cells along with K^+^.	IV push of 50 mEq. The effect appears 15–30 min and lasts 2–6 h	Hypernatremia Volume overload
Furosemide: Delayed response and inconsistent effect	Elimination of K^+^ from the body.	Furosemide 40–80 mg IV (large doses may be needed in renal failure). The effect appears within 5–30 min and lasts 2–6 h.	Hypotension Worsening renal function

ECG: electrocardiogram.

## Data Availability

Not applicable.
